# Overcoming antibiotic resistance: non-thermal plasma and antibiotics combination inhibits important pathogens

**DOI:** 10.1093/femspd/ftae007

**Published:** 2024-05-10

**Authors:** Eva Vaňková, Jaroslav Julák, Anna Machková, Klára Obrová, Anja Klančnik, Sonja Smole Možina, Vladimír Scholtz

**Affiliations:** Department of Physics and Measurements, University of Chemistry and Technology in Prague, 160 00 Prague, Czech Republic; Department of Biotechnology, University of Chemistry and Technology in Prague, 160 00 Prague, Czech Republic; Department of Physics and Measurements, University of Chemistry and Technology in Prague, 160 00 Prague, Czech Republic; Institute of Immunology and Microbiology, First Faculty of Medicine, Charles University in Prague, 160 00 Prague, Czech Republic; Department of Physics and Measurements, University of Chemistry and Technology in Prague, 160 00 Prague, Czech Republic; Department of Physics and Measurements, University of Chemistry and Technology in Prague, 160 00 Prague, Czech Republic; Department of Food Science and Technology, Biotechnical Faculty, University of Ljubljana, 1000 Ljubljana, Slovenia; Department of Food Science and Technology, Biotechnical Faculty, University of Ljubljana, 1000 Ljubljana, Slovenia; Department of Physics and Measurements, University of Chemistry and Technology in Prague, 160 00 Prague, Czech Republic

**Keywords:** antimicrobial resistance (AMR), cold atmospheric plasma (CAP), combinatory therapy, methicillin-resistant *Staphylococcus aureus*, *Pseudomonas aeruginosa*

## Abstract

Antibiotic resistance (ATBR) is increasing every year as the overuse of antibiotics (ATBs) and the lack of newly emerging antimicrobial agents lead to an efficient pathogen escape from ATBs action. This trend is alarming and the World Health Organization warned in 2021 that ATBR could become the leading cause of death worldwide by 2050. The development of novel ATBs is not fast enough considering the situation, and alternative strategies are therefore urgently required. One such alternative may be the use of non-thermal plasma (NTP), a well-established antimicrobial agent actively used in a growing number of medical fields. Despite its efficiency, NTP alone is not always sufficient to completely eliminate pathogens. However, NTP combined with ATBs is more potent and evidence has been emerging over the last few years proving this is a robust and highly effective strategy to fight resistant pathogens. This minireview summarizes experimental research addressing the potential of the NTP-ATBs combination, particularly for inhibiting planktonic and biofilm growth and treating infections in mouse models caused by methicillin-resistant *Staphylococcus aureus* or *Pseudomonas aeruginosa*. The published studies highlight this combination as a promising solution to emerging ATBR, and further research is therefore highly desirable.

## Teaser

The use of a combination of non-thermal plasma and antibiotics against dangerous pathogens is a potential solution to the antimicrobial resistance crisis.

## Introduction

Antimicrobial resistance (AMR), often referred to as the “silent pandemic”, impacts the treatment of infections less obviously than “visible” health crises, leading to higher healthcare costs, longer hospital stays and increased mortality. Its impact extends beyond healthcare, also affecting agriculture and ecology (Schnall et al. 2019). In this minireview, we focus on a novel approach to tackling the emergence of antibiotic resistance (ATBR) and multidrug-resistant (MDR) bacteria. We discuss factors contributing to the emergence of ATBR and MDR pathogens and introduce the favorable properties of non-thermal plasma (NTP) and its potential for use in medicine, particularly in treating infections caused by resistant pathogens. We summarize experimental studies combining NTP pre-treatment and antibiotics (ATBs) to enhance inhibition efficacy, primarily against typically used model representatives of gram-negative and gram-positive bacteria, *Pseudomonas aeruginosa* and methicillin-resistant *Staphylococcus aureus* (MRSA), respectively. A growing body of evidence shows that NTP has real potential for mitigating ATBR and restoring the sensitivity of MDR pathogens in medical practice and infection treatment.

## Antibiotic resistance: a worldwide threat

ATBR, a global health problem of the 21st century, is caused by the rise of MDR pathogens and the lack of novel treatment approaches. If no action is taken, MDR microorganisms will become the leading cause of death worldwide by 2050 (WHO 2021; Tang et al. 2023). ATBR increases the global health problems rate, mortality and healthcare costs because of the failure of ATBs to eliminate common infections (Botelho et al. 2019, Gajdács et al. 2021). In addition, high costs and lengthy procedures slow down the development of novel ATBs, further exacerbating the ATBR crisis (Daikos et al. 2021, Gajdács et al. 2021).

### Emergence of ATBR

The ATBR crisis is primarily caused by the overuse of ATBs in healthcare, agriculture and animal husbandry. Misuse in prescribing ATBs for viral infections and poor patient compliance with prescribed dosing contribute to ATBR development (WHO 2021). In animal husbandry, misuse of ATBs leads to the emergence of MDR bacteria potentially transmittable to humans. The ATBR emergence is exacerbated by additional factors, such as improper disposal of ATBs, ATBs treatment discontinuation, ATBs being available without medical prescription, as well as inconsistent global regulations (Polianciuc et al. 2020). To tackle this crisis, stricter regulations, better diagnostics, greater public awareness and investments in new treatment approaches, as well as research, are required.

The European Union summary report, issued by the European Food Safety Authority (EFSA) and the European Centre for Disease Prevention and Control (ECDC), tracks AMR trends in bacteria isolated from animals, food of animal origin and humans in 2019–2020 (EFSA 2022) and shows an alarming increase in AMR. These trends of increasing ATBR in common bacteria highlight the need for continued surveillance, research and collaboration at European and global levels.

### Current status of MDR pathogens

According to recent estimates, ∼4.95 million deaths were associated with bacterial AMR in 2019 (Lancet 2022). Although in 2017, the World Health Organization (WHO) list of “priority pathogens” included the ESKAPE pathogens—*Enterococcus faecium, Staphylococcus aureus, Klebsiella pneumoniae, Acinetobacter baumannii, P. aeruginosa* and *Enterobacter* spp. (WHO 2017, De Oliveira et al. 2020, Mancuso et al. 2021, Teng et al. 2023)—pathogens such as *Neisseria gonorrhoeae, Mycobacterium tuberculosis* and *Candida* spp. are becoming increasingly resistant as well, reflecting a general trend towards AMR (Ventola 2015). The resistance of important pathogens to conventionally used ATBs is summarized in Table 1.

**Table 1.** Summary of resistance of important pathogens to conventionally used antibiotics (ATBs).

Bacteria	ATBs	Level of resistance	Reference
*Staphylococcus aureus*	penicillin	74%	Congdon et al. (2023)
*S. aureus*	azithromycin	34%	Congdon et al. (2023)
*S. aureus*	cefoxitin	5%	Congdon et al. (2023)
*S. aureus*	ciprofloxacin	5%	Congdon et al. (2023)
*S. aureus*	tetracycline	4%	Congdon et al. (2023)
*S. aureus*	trimethoprim	1%	Congdon et al. (2023)
*Pseudomonas aeruginosa*	carbapenems	20%–50%	ECDC, 2023
*Escherichia coli*	cephalosporins	10%–30%	WHO, 2022
*E. coli*	ampicillin, co-trimoxazole, fluoroquinolones	over 20%	ECDC, 2023
*E. coli* (UTI)	ciprofloxacin	8%–65%	WHO, 2022
*Klebsiella pneumoniae*	various ATBs	over 57%	WHO, 2022
*K. pneumoniae*	carbapenems	over 50%	WHO, 2022
*Acinetobacter* spp.	various ATBs	over 56%	WHO, 2022
*Acinetobacter baumannii*	various ATBs	over 50%	ECDC, 2023
*Shigella* spp.	ampicillin	over 30%	CDC, 2023
*Enterococcus* spp.	vancomycin	over 30%	CDC, 2019a
*Clostridium difficile*	vancomycin	4.7%	Boyanova et al. (2023)
*C. difficile*	metronidazole	2.6%	Boyanova et al. (2023)
*C. difficile*	moxifloxacin	34.9%	Boyanova et al. (2023)
*C. difficile*	clindamycin	61%	Boyanova et al. (2023)
*C. difficile*	erythromycin	60%–88%	Boyanova et al. (2023)
*C. difficile*	rifampicin	23%–55%	Boyanova et al. (2023)
*C. difficile*	tigecycline	0%–5%	Boyanova et al. (2023)
*C. difficile*	fidaxomicin	0%–2%	Boyanova et al. (2023)
*Mycobacterium tuberculosis*	isoniazid, rifampicin	5%	CIDRAP, 2017
*Mycoplasma genitalium*	macrolides	over 50%	Wood et al. (2023)
*Streptococcus pneumoniae*	macrolides	39.9%	Mohanty et al. (2023)
*S. pneumoniae*	penicillin	39.6%	Mohanty et al. (2023)
*Campylobacter* spp.	fluoroquinolones	over 20%	CDC, 2023
*Campylobacter jejuni*	ciprofloxacin	28%	CDC, 2019b
*C. jejuni*	azithromycin	3%	CDC, 2019b
*Campylobacter coli*	ciprofloxacin	38%	CDC, 2019b
*C. coli*	azithromycin	7%	CDC, 2019b
*Neisseria gonorrhoea*	ciprofloxacin	over 60%	ARSE, 2023
Various bacteria	penicillin	0%–51%	WHO, 2022

The search for alternatives to tackle AMR typically employs MDR strains of MRSA and *P. aeruginosa*. MRSA alone was reported to cause more than 100 000 deaths across 204 countries in 2019 (Wu et al. 2019, Guo et al. 2020), and the treatment of its infections is a challenge due to ATBR (Wu et al. 2019). Altered penicillin-binding proteins grant MRSA strains resistance to methicillin and other β-lactam ATBs (Gajdács 2019) as well as vancomycin (Haseeb et al. 2019, Guo et al. 2020). Mutations in the *rpoB* gene, associated with impaired membrane permeability, as well as increased biofilm formation and reactive oxygen species production, led to rifampicin resistance (Portelli et al. 2020, Zhang et al. 2023a). Resistance to trimethoprim-sulfamethoxazole, used for MRSA treatment, has also been reported (Nurjadi et al. 2022, Ham et al. 2023). Similarly, resistance to ampicillin and kanamycin, partially reversible by metallic micronutrients, has been reported for MRSA (Garza-Cervantes et al. 2020). Mutations in the *mecA* gene have been linked to oxacillin-resistance (Goering et al. 2019). More pronounced ATBR in MRSA biofilm compared with the planktonic form has been reported (Craft et al. 2019, Guo et al. 2020, Gajdács et al. 2021).

*P. aeruginosa* typically infects immunocompromised individuals, for example, patients after invasive surgeries, with diabetes, or with cystic fibrosis (Botelho et al. 2019, Qin et al. 2022). Its ability to mutate rapidly and transfer genes contributes to ATBR, especially to aminoglycosides, quinolones and β-lactams, making treatment difficult (Wang et al. 2020, Qin et al. 2022). Efflux pumps and altered cell membranes were reported as the MDR mechanism of *P. aeruginosa* to chlorhexidine (Cieplik et al. 2019, Wang et al. 2020, Tag ElDein et al. 2021). The PhoP-PhoQ two-component regulatory system contributes to the resistance to polymyxin B, the last treatment option typically used against MDR gram-negative bacteria (Yang et al. 2021a). Biofilm formation also increases ATBR in this bacterium. Data indicate a correlation between the occurrence of MDR bacteria, biofilm formation and the expression of virulence, underlining the role of *P. aeruginosa* as a key model in the study of biofilm-related ATBR (Eladawy et al. 2021, Gajdács et al. 2021, Silva et al. 2023).

## NTP: a powerful multi-purpose tool

Plasma is a partially or fully ionized gas that can exhibit highly variable properties in terms of temperature (millions of Kelvins to room temperature), pressure and composition, depending on its source. NTP has found application in many fields, for example, modification and functionalization in material science, environmental science and agriculture, food industry or biological and medical applications (Murugesan et al. 2020, Barjasteh et al. 2021, Asl et al. 2022, Moszczyńska et al. 2023).

The following sections focus on NTP, also known as cold atmospheric plasma, generated at atmospheric pressure. Its ambient temperature is gentle to the treated material, making it well suited for medical applications, for example, as a novel non-ATB antimicrobial treatment (Moreau et al. 2008, Brun et al. 2018). In addition, NTP can be used to treat the surfaces of various materials, which then have antibacterial or self-cleaning properties (Mozetič 2019).

### Medical fields benefitting from NTP treatment

Plasma medicine is a new field that was founded ∼25 years ago, and that successfully implemented the use of NTP-generated reactive oxygen and nitrogen species (RONS) for medical targets, benefitting from their antimicrobial effect (Laroussi 2020), and also yielding a clinical trial on NTP-mediated wound healing in 2010 with encouraging results (Isbary et al. 2010). In addition to wound healing, NTP has applications in dermatology, particularly for inflammatory skin irritations (Heinlin et al. 2011, Emmert et al. 2013, Wirtz et al. 2018), skin infections (e.g. mycosis, onychomycosis, acne-prone skin) (Chutsirimongkol et al. 2014, Lux et al. 2020), abscesses, burns or the removal of scars and skin growths, among others (Bernhardt et al. 2019). Furthermore, NTP has been successfully used in ophthalmology (Reitberger et al. 2018), in dentistry (Pan et al. 2013, Dong et al. 2014, Delben et al. 2016, Gherardi et al. 2018), where the AMR of dental biofilm bacteria is becoming an urgent problem, and in orthopedics, for treating post-surgical infections (Nguyen et al. 2018). NTP might have applications in regenerative medicine and medical engineering due to its influence on stem cell proliferation (Miletić et al. 2013, Park et al. 2016, Alemi et al. 2019, Xiong et al. 2019). NTP-mediated decontamination of medical instruments and other equipment benefits from the antimicrobial properties of NTP, and its efficiency against biofilms and MDR pathogens, as demonstrated by NTP-mediated biofilm removal from endotracheal tubes with possible application for other endoscopes (İbiş and Ercan 2020).

Finely tuned doses of NTP have been shown to selectively kill cancer cells without harming healthy cells, opening a new field of plasma oncology being tested for leukemia, carcinoma, breast cancer, brain cancer, prostate cancer, colorectal cancer and others (Laroussi 2014, Yan et al. 2021). Preliminary studies in Germany used NTP in palliative therapy in pain mitigation of ulcerations for head and neck cancer patients (Metelmann et al. 2015). The efforts described above eventually culminated in the US Food and Drug Administration approving the first American oncological clinical trial in 2019. Moreover, NTP application may not remain limited to surfaces, as miniaturized microplasma devices for subcutaneous and internal application are currently under development. For example, NTP was successfully used for nasal mucosal regeneration *in vitro* and *in vivo* (Won et al. 2018) and the regeneration of several nerve cell types isolated from animal models (Katiyar et al. 2019).

An important implication of plasma medicine is the efficient inactivation of dangerous resistant pathogens, with ESKAPE being a primary target (Scholtz et al. 2021). *In vivo* and *ex vivo* experiments provide evidence for NTP-mediated inhibition of *S. aureus, E. coli, P. aeruginosa* and *A. baumannii* (Li et al. 2023), as well as promoted healing of local burns, accompanied by good biological acceptance, only mild adverse reactions and overall shortening of the course of treatment. NTP treatment of experimentally wounded and MRSA-infected rabbits led to a decrease in cytokine secretion, inflammatory response and immune cell proliferation, and to accelerated re-epithelialization and wound healing (Li et al. 2021a). NTP-mediated decrease of *P. aeruginosa* load and biofilm formation was demonstrated in wounds of diabetic mice (Cooley et al. 2020) and in a human skin wound model in a recent preclinical study of burns healing (Bagheri et al. 2023).

Several certified NTP-generating devices for the treatment of surface infections are currently on the market. The kINPen® MED is the first CE-certified class IIa medical device for the treatment of chronic wounds and pathogen-induced skin disorders. PlasmaDerm® VU-2010 (CINOGY Technologies GmbH, Duderstadt, Germany; CE-certified in Germany by MEDCERT, ISO 13485) and SteriPlas (Adtec Ltd., London, UK) are certified for the activation of chronic and acute wound healing by changing its microenvironment and reducing the microbial load, as well as of MDR pathogens. The Jett Plasma devices (COMPEX, s.r.o, Brno, Czech Republic) are certified for dermatology, aesthetic medicine and ophthalmology.

### Generation and favorable properties of NTP

NTP is typically generated by supplying ionization energy to gas using, for example, an electrical discharge. Its macroscopic temperature is ambient or slightly higher (typically not more than 40°C), but highly energetic electrons induce a rich mixture of different reactive particles (light electrons have significantly higher energy than heavy ions and neutral particles). The chemical composition of NTP depends on its generation parameters, including applied voltage and feeding gas. Ambient air is commonly used, but other gases like Ar, He or their mixture with O_2_ are used for special applications (Tendero et al. 2006). RONS are generated in NTP either directly from O_2_ and N_2_ in air, or from particles (e.g. He, Ar) interacting with biological material. The most effective antimicrobial RONS are hydroxyl radical OH·, atomic oxygen O·, singlet oxygen ^1^O_2_, superoxide radical O_2_^−^, atomic nitrogen N and excited states of N_2_ and NO*_x_*. These active species interact with living cells, which is also the reason for the proven antimicrobial efficacy of NTP (Graves 2012).

The advantage of NTP is its antimicrobial activity against MDR bacteria, while not inducing primary or acquired resistance (Zimmermann et al. 2012). This is due to the mechanism of NTP action, primarily oxidative stress induction by RONS, leading to cell membrane rupture, cytoplasmic leakage and degradation of intracellular components (Kartaschew et al. 2016, Ma et al. 2022). Therefore, NTP is unlikely to act with different efficiency on MDR and non-resistant bacterial strains (Sakudo and Misawa 2020). Furthermore, NTP-mediated inhibition of conjugative transfer of resistance genes, and thus inhibition of the very emergence of ATBR, has been reported (Li et al. 2021b). Generally, the antimicrobial effect of NTP is mediated by a combination of several mechanisms that have not yet been fully elucidated. The specific impact and importance of each mechanism also depends on the type of NTP-generating device used (Tendero et al. 2006, Ehlbeck et al. 2010, Šimončicová et al. 2019), but the following are typically present (Graves 2012, Scholtz et al. 2021): lipid peroxidation and protein denaturation both in membranes and in cytoplasm, triggering of metabolic and apoptotic pathways by RONS (Čtvrtečková et al. 2019, Akter et al. 2020), accumulation of charged particles on the cell surface, UV radiation (only marginal for most plasma sources) (Machala et al. 2010) and etching (Moisan et al. 2002, Zhang et al. 2023b). Most of these mechanisms target functional cellular pathways that are potentially susceptible to escape mutations granting resistance. However, the etching (at higher NTP doses) additionally causes physical damage to the membrane, eventually leading to its rupture. This is very interesting and important as cells are unable to cope with physical damage using their usual defense mechanisms, including the emergence of resistance. Certain bacterial cell forms, such as endospores or biofilms, are covered by polysaccharide-protective layers efficient against environmental threats, typically the immune system. Although granting some protection, these layers do not provide resistance to NTP-mediated damage (Julák et al. 2020, Paldrychová et al. 2020, Khosravi et al. 2021, Das et al. 2022, Liu et al. 2022).

NTP treatment can be applied indirectly as well, when applied to a liquid (water, saline or others), which accumulates reactive particles and can be used for applications at a later time or different place. This phenomenon of plasma-activated water (PAW) or plasma-activated saline (PAS) has already been described (Machala et al. 2018, Zhou et al. 2020). Nevertheless, the direct physical damage and membrane etching mentioned above are only very limited upon the application of PAW or PAS.

## Combination of NTP pre-treatment and ATBs action

To date, there are very few publications addressing the use of a NTP-ATBs combination for overcoming the ATBR of dangerous pathogens. With one exception, all the evidence regarding the NTP enhancement of conventional ATBs, mainly in the treatment of infections caused by MRSA and *P. aeruginosa*, has only emerged since 2020. Because the published research addressing the combination is still very limited, to date, the mechanisms of the synergy have not been the focus. However, we can speculate that NTP capable of damaging the cell by a combined mechanism of action leaves the bacterium exhausted and unable to counter the ATB-mediated pressure. At the same time, NTP causes the release of free planktonic cells from resistant biofilm structures, leading to increased sensitivity to ATBs action. While individual ATBs target specific pathways in cells, the broadly acting NTP is unlikely to do that. This is crucial for the synergistic effects on MDR bacteria. ATBR is metabolically and energetically demanding and encourages the bacterium to invest resources in escaping the respective agent, leaving insufficient resources to tackle NTP-induced damage. However, given that each ATB specifically targets specific pathways in cells, it is impossible to anticipate a common mechanism of action without detailed experimental studies, as it may be different for each ATB used in combination with NTP. Moreover, ATBR metabolic pathways do not increase resistance to NTP and thus NTP and ATBs can act as completely independent agents. NTP is sufficiently gentle for live tissue applications, but due to their sensitivity, a synergy of two different approaches is beneficial. Such a synergistic combination could provide benefits to the treated tissue, reduce the environmental and health impact of ATB use and also increase the efficacy of ATBs against MDR bacteria, thus overcoming the ATBR problem.

As mentioned above, the antimicrobial effect of NTP is highly dependent on the parameters of its generation. Therefore, detailed NTP properties, as well as ATB concentrations used in existing experimental studies and the resulting antimicrobial/antibiofilm effects, are summarized in Table 2.

**Table 2.** Detailed summary of information reported in experimental studies describing the use of a combination of non-thermal plasma and antibiotics in inhibiting important pathogens.

Bacteria	NTP source				ATB	Order of treatment in combination	Treated sample	Results			Reference
	Discharge	Voltage	Frequency	Gas				NTP exposure time	Concentration of ATB	Effectivity	
Methicillin-resistant *Staphylococcus aureus* (MRSA)	pulsed DBD	5 kV	-	air	trimethoprim	NTP pre-treatment	surface growth on TSB agar	10 or 30 s	-	moderate	Bayliss et al. (2013)
					kanamycin				-	weaker	
					oxacillin				-	weak	
					norfloxacin				-	none	
MRSA	AC surface discharge	7.44 kV	20 kHz	air/helium	rifampicin	NTP pre-treatment	biofilm on TSB; mouse wound infection model (MWIM)	2–6 min (BC), 6 min once for 3 days (MWIM)	625 mg/L (BC); 30 mg/kg (MWIM)	high (BC), high (MWIM)	Guo et al. (2021)
					ciprofloxacin				1250 mg/L (BC)	high	
					norfloxacin				1250 mg/L (BC)	high	
					vancomycin				1250 mg/L (BC)	high	
MRSA	Plasma activated saline (PAS)	-	-	-	vancomycin	PAS pre-treatment	biofilm on silica films; mouse systemic infection model (MSIM)	30 min cultivation with PAS (BC), intraperitoneal injection of PAS once for 5 days	0.625–1.25 g/L (BC)	high (BC)	Yang et al. (2021b)
					rifampicin				0.3125–0.625 g/L (BC), 30 mg/kg (MSIM)	high (BC, MSIM)	
*P. aeruginosa* (different strains)	DC cometary discharge	5 kV	-	air	gentamicin	NTP pre-treatment	biofilm on Ti-6Al-4V	15–60 min based on strain used	4–9 mg/L	generally high	Paldrychová et al. (2020)
					ceftazidime				0.1–1 mg/L		
					polymyxin B				3.5–15 mg/L		
*P. aeruginosa*	AC DBD	8 kV	50 kHz	air	ciprofloxacin	NTP pre-treatment	biofilm in 96-well MTP or stainless-steel coupons	3 min	0–100 mg/L	high	Muraca et al. (2021)
*P. aeruginosa*	AC plasma jet	9 kV	22 kHz	argon	ciprofloxacin	NTP pre-treatment	biofilm in 6-well MTP	5–90 s	16 mg/L	moderate	Khosravi et al. (2022)
*P. aeruginosa* (different strains)	AC DBD plasma jet	6 kV	20 kHz	helium/oxygen	gentamicin	NTP pre-treatment	planktonic cells (PC) and biofilm cells (BC) in Calgary device	45 s for PC, 90 s for BC	0.125–16 mg/L (PC), 0.5–256 mg/L (BC)	moderate	Maybin et al. (2023)
					tobramycin				0.25–16 mg/L (PC), 0.5–128 mg/L (BC)	highest	
					ciprofloxacin				0.0313–5 mg/L (PC), 0.125–64 mg/L (BC)	moderate	
					disinfection chlorhexidine				11.25–90 mg/L (PC), 11.25–1440 mg/L (BC)	lowest	
*Staphylococcus epidermidis*	DC cometary discharge	5 kV	-	air	erythromycin	NTP pre-treatment	biofilm on Ti-6Al-4V	15 min	10 mg/L	high	Julák et al. (2020)
*Escherichia coli*					polymyxin B			30 min	15 mg/L	moderate	
*Candida albicans*					amphotericin B			30 min	2.5 mg/L	weak	
*Morganella* spp.	AC DBD	65 kV	50 kHz	-	antimicrobial peptide bacteriocin (Lactocin C-M2)	C-M2 application prior NTP treatment	planktonic cells	120 s	300 mg/L	high	Shan et al. (2020)
*Listeria innocua*	AC DBD	7 kV	15 kHz	helium/oxygen	bacteriocin nisin	variation in order of treatment	planktonic cells or surface growth on xanthan gum gel	0–30 min	35 IU/mL	high when nisin applied before NTP exposure	Costello et al. (2021)

Abbreviations: AC, alternating current; ATB, antibiotic; DBD, dielectric barrier discharge; DC, direct current; MTP, microtiter plate; NTP, non-thermal plasma.

### NTP-ATBs combination targeting MRSA *in vitro*

The very first mention, to the best of our knowledge, of combining NTP pre-treatment with ATB action was published in 2013 (Bayliss et al. 2013). This important pioneering report was published in the form of a Letter to the Editor and therefore contained very limited information. Nevertheless, its success made the authors propose the combination therapy for post-operative infections, burns or leg and foot ulcers. They tested NTP pre-treatment of MRSA (undefined strain) cultures on Tryptone Soya Broth (TSB) agar followed by application of ATB test strips with trimethoprim, kanamycin, oxacillin or norfloxacin. Regained ATB sensitivity, demonstrated by a clear inhibition zone, was detected after 10 s of NTP pre-treatment combined with trimethoprim, and after 30 s of NTP combined with the other ATBs. Although the inhibition was not complete, as demonstrated by several isolated bacterial colonies within the inhibition zones, the results showed that NTP pre-treatment is capable of reversing resistance to certain ATBs.

An enhanced antibiofilm effect of NTP pre-treatment followed by ATBs (rifampicin, ciprofloxacin, norfloxacin and vancomycin) against MRSA was reported (Guo et al. 2021). MRSA ATCC 33591 biofilm formed on TSB agar plates was treated with NTP for 2, 4 or 6 min and subsequently with ATBs at concentrations of 625 (rifampicin) and 1250 mg/L (other ATBs), respectively. It was shown that NTP treatment enhanced the effect of rifampicin on the reduction of MRSA biofilm.

PAS demonstrated a synergistic effect with ATB action against MRSA biofilm (Yang et al. 2021b). When MRSA was incubated in the presence of PAS for 30 min and combined with vancomycin (1.25 and 0.625 g/L for 24 h) or rifampicin (0.625 and 0.3125 g/L for 24 h), PAS reduced MRSA ATCC 33591 biofilm *in vitro* by at least six orders of magnitude colony forming units per milliliter (CFU/mL), while PAS, vancomycin and rifampicin alone only reached 1.2, 1.2 and 3.6 orders of magnitude, respectively.

### NTP-ATBs combination targeting resistant *Pseudomonas aeruginosa in vitro*

Improved ATB action and even eradication of *P. aeruginosa* biofilms upon NTP treatment was reported by Paldrychová et al. (2020). Four strains of *P. aeruginosa* (DBM 3081 and 3777, ATCC 10145 and 15442) were exposed to NTP (15–60 min) and cultured in subinhibitory doses of gentamicin, ceftazidime and polymyxin B. The susceptibility of individual strains to NTP, ATBs and their combination differed a lot, thus the right set-up needs to be determined for each strain; however, in general, NTP induced a higher ATB susceptibility, with gentamicin requiring the lowest concentrations (4–9 mg/L) for inhibition. A complete eradication of mature *P. aeruginosa* ATCC 15442 biofilm from Ti-6Al-4 V orthopedic alloy was achieved after 15 min of NTP and 8.5 mg/L gentamicin combination treatment, as shown by scanning electron microscopy (SEM).

The effect of ciprofloxacin against *P. aeruginosa* PAO1 planktonic cells and biofilm can be boosted with NTP pre-treatment (Muraca et al. 2021). The ciprofloxacin MIC of biofilm (200 mg/L) was reduced by one-half upon NTP pre-treatment (50–100 mg/L). SEM visualization showed that despite considerable inhibition, complete eradication of biofilm was not achieved after exposure to NTP for 3 min followed by ciprofloxacin. As residual cells and the risk of re-infection are not acceptable in medical practice, conditions yielding complete eradication should be determined first. This study also addressed nanostructured lipid carriers delivery of ciprofloxacin. Although the efficacy of ciprofloxacin against *P. aeruginosa* biofilm was enhanced in this formulation, no synergistic effect was achieved when combined with NTP.

Ciprofloxacin was also used by Khosravi et al. (2022), who investigated NTP pre-treatment of MDR *P. aeruginosa* (isolated from clinical specimens) biofilm followed by subinhibitory concentration of the ATB (16 mg/L). While ciprofloxacin inhibited biofilm biomass and cell viability by ∼70%, 90 s of NTP treatment led to ∼85% inhibition. Moreover, SEM visualization showed that bacterial cells in the biofilm lysed and mostly only cell debris and extracellular polymers remained on the surface of carrier; there were no intact viable bacterial cells. The presence of cell debris can bias conventional biofilm quantification assays, and indeed, fluorescence microscopy showed a substantial reduction in the biofilm biomass, with only a few residual cells still on the carrier surface. These results are very promising, even although complete inhibition will require more stringent conditions.

A recent study that employed NTP in combination with ciprofloxacin, as well as other ATBs (gentamicin and tobramycin compared with disinfectant chlorhexidine) (Maybin et al. 2023), also reported that NTP pre-treatment increases the susceptibility of both planktonic and biofilm cells of *P. aeruginosa* (strains PAO1, PA14 and PA10548) to subinhibitory concentrations of ATBs. In addition to standard methods like CFU/mL counting and metabolic activity determination (isothermal microcalorimetry), a number of methods (e.g. transcriptomic analysis, signaling molecules tracking with hyperphosphorylated guanosine, and detection of extracellular ATP and LDH) were used to address the mechanism of action. The highest enhancement of ATB action by NTP pre-treatment was exhibited for tobramycin (biofilm-eradicating concentration dropping impressively from 256 to 2 mg/L after 90 s of NTP), followed by gentamicin and ciprofloxacin, and the lowest enhancement efficacy was observed for the disinfectant chlorhexidine. NTP enhanced ATB action in terms of cell metabolic activity inhibition in all tested cases. Transcriptomic analysis showed activation of pathways mitigating NTP-mediated oxidative stress (discussed in chapter Generation and favorable properties of NTP), for example, peroxide dismutase, oxidases, catalases, peroxidases and denitrification genes. Moreover, a switch from biofilm to planktonic cells was detected 6 h after exposure, as ribosome modulation factor, involved in the formation of persistent cells, was downregulated. This finding is consistent with previously published (Kašparová et al. 2022) NTP-mediated release of *P. aeruginosa* cells from biofilm to their planktonic form. Overall, Maybin et al. (2023), similar to the other studies discussed in this section, concluded that NTP pre-treatment can be an effective strategy for restoring the susceptibility of *P. aeruginosa* biofilms to antimicrobial agents.

### NTP-ATBs combination targeting other dangerous bacterial pathogens *in vitro*

The pioneering publication describing the NTP and ATBs combination against biofilm (Julák et al. 2020) used ATB-resistant bacteria *Staphylococcus epidermidis* and *Escherichia coli*, and a yeast *Candida albicans*. The biofilms were treated with NTP (15 and 30 min) and subsequently with erythromycin (10 mg/L), polymyxin B (15 mg/L) and amphotericin B (2.5 mg/L), respectively. While fluorescence microscopy showed a reduction of biofilm area in all cases, only the bacteria showed an enhanced reduction in metabolic activity upon the combination treatment. Importantly, the exposure to NTP and ATBs resulted in an efficient prevention of biofilm re-development from persistent cells, which makes this combination therapy a promising strategy in the treatment of pathogens.

A unique study reported a synergy between NTP treatment and an antimicrobial peptide bacteriocin (Lactocin C-M2) used against putrefactive bacteria *Morganella* spp. wf-1 isolated from aquatic foods (Shan et al. 2020). The middle-tested concentration (0.3 g/L) of Lactocin C-M2 combined with 90 s of NTP exposure synergistically reached a decrease in *Morganella* spp. cells by ∼6-fold. Transmission electron microscopy of bacteria treated with the combination revealed disruption of cell membranes, cytoplasmic condensation, DNA relaxation, abnormal septation, irregular cross-wall formation and even cellular lysis of greater magnitude than when treated with Lactocin C-M2 or NTP alone. In addition, the combination treatment led to a higher leakage of K+, phosphates, DNA/RNA, proteins and enzymes than after Lactocin C-M2 or NTP alone.

Food decontamination with NTP and nisin, a natural bacteriocin produced by lactic acid bacteria, was tested using *Listeria innocua* grown planktonically or on the surface of xanthan gum gel (Costello et al. 2021). Four different arrangements of bacterial cells were tested: grown in a liquid food product; grown in water used to wash a solid food product; grown on the surface of a food product; and grown on one solid product and transferred to another product. For nisin treatment, 50 µL of 35 IU/ml solution was dropped onto the surface of the culture disc and followed by NTP treatment for 30 min. The combination was more effective than individual treatments, but only when nisin was applied prior to NTP. The study also provided insights into the environmental stress response and adaptation of *L. innocua* grown in structured systems to natural antimicrobials and novel antimicrobial technologies, and represents a step towards application of food-decontamination methods in the food industry.

### NTP-ATBs combination targeting dangerous pathogens *in vivo* and its possible clinical applications

The studies described in the previous section demonstrated synergy between NTP and ATBs *in vitro*. Given the novelty of this approach in both plasma medicine and ATBR prevention, only limited data have demonstrated synergy *in vivo*. However, two studies performed in mouse models support the *in vitro* findings and highlight the potential for implementing this technology in clinical practice.

One of the above-mentioned studies (Guo et al. 2021) addressed not only the *in vitro* effect, but also the treatment of MRSA-infected wounds in a mouse model. Shaven and disinfected mice were wounded under anesthesia and infected with MRSA. Over 3 days thereafter, posterior parts of the infected mice were treated with NTP for 6 min once a day, and rifampicin (30 mg/kg) was administered intragastrically every 12 h. The number of bacteria counted *in vitro* as well as from sacrificed mice clearly showed that NTP treatment enhanced the effect of rifampicin on the reduction of MRSA biofilm. Moreover, blood and histochemical analyses demonstrated a favorable biosafety profile of the combined treatment.

In the second study, PAS demonstrated a synergistic effect with ATB action also against MRSA for systemic infection studies. Mice were inoculated with 100 µL of MRSA suspension into the lateral tail vein, inducing systemic infection but not death during the experimental cycle. From day 2 to day 4 post infection, 150 µL of PAS was administered intraperitoneally and 30 mg/kg rifampicin was administered intragastrically once a day. On day 5, blood was sampled from an eye vein, mice were sacrificed and their guts tested for bacteria presence. PAS combined with rifampicin synergistically and effectively reduced the MRSA infection (as opposed to non-combination therapy) investigated by histology, as well as improved hematological and biochemical parameters of infected mice.

For clinical applications of NTP, a number of obvious problems need to be addressed (e.g. the treatment of systemic infections). Superficial infections can be readily tackled with the combination of surface NTP application and systemic or topical ATB administration. In such cases, NTP can be specifically and precisely targeted onto infected tissue and the treatment enhanced by ATB application. Nevertheless, the above-discussed study using indirect application of NTP in the form of PAS, which can be administered systemically, suggests a way of addressing systemic infections in the future. However, a detailed discussion of this approach is, considering the single published study to date, beyond the scope of our speculations.

Unlike the systemic administration, the topical external application of the NTP-ATBs combination in clinical practice, for example, against bacterial infections such as acne, atopy, abscesses, wound infections, military wounds and burns, is clearly a promising idea. Our study of mycotic skin infections showed a significant therapeutic effect of the simultaneous administration of systemic antifungal agents with external application of NTP (Lux et al. 2020). A similar success can be expected when the same approach is applied to MDR bacteria. In our opinion, the treatment of non-systemic infections with a NTP-ATBs combination has major therapeutic potential, which should be translated into clinical trials as well.

Another promising application is in bacterial bladder infections. As an internal infection, this requires a more complex approach, and could benefit from the promising results with PAS. PAS of compositions harmless to the bladder epithelium could be supplemented directly into the bladder, which would restore the ATBs sensitivity of infecting MDR bacteria. Alternatively, direct administration of NTP via a catheter comes into consideration. It is worth noting that electrocoagulation, a method used in clinical practice for a long time, has a very similar generation principle to NTP. However, preliminary studies mapping the effect of PAS on the bladder epithelium, as well as *in vitro* or *in vivo* testing of PAS and NTP effects on MDR bacteria responsible for bladder infections, have yet to be conducted.

The two visions discussed above appear to be best suited for initial use, as other complex or systemic infections are likely to be more difficult to treat. Treatment of internal infections of a known origin may resemble the treatment of cancer, for which the application of NTP has been studied for a long time (Partecke et al. 2012, Bekeschus 2023). However, clinical evidence of successful gas NTP application in cancer patients is rare and no malignancy treatment involving gas NTP has been established in clinical practice to date.

Additionally, NTP application to mitigate contamination during implant placement may also be a promising possibility. We addressed this option by *in vitro* studies (Paldrychová et al. 2020) of biofilm elimination from Ti-6Al-4 V alloy used in orthopedics using a NTP-ATBs combination. During implantation placement, an appropriate exposure to NTP could reduce the bacteria load or increase bacteria susceptibility to ATB prophylaxis. Implant materials are generally highly resistant and therefore NTP could be applied in much higher doses than typically used for sensitive tissues. Even this *ex vivo* preventive application of NTP could significantly reduce the risk of subsequent infection.

## Conclusion

AMR and ATBR represent a global health problem of major importance. MDR pathogens are emerging across bacterial strains, ATBR to most medically used ATBs has been reported and novel ATB development is not keeping up with the pace. Tackling the complex AMR/ATBR challenge requires a coordinated global effort that includes improved surveillance, enhanced infection control, prudent use of ATBs in human and veterinary medicine, as well as increased investment in research into alternative therapies and innovative strategies. We believe that NTP represents a promising tool to either combat MDR pathogens directly or to restore their ATB susceptibility and regain control over the treatment. NTP alone is capable of inactivating bacteria and the specificities of its action minimize the risk of inducing resistance. However, synergy between NTP and ATBs achieves much better results, as demonstrated by a number of *in vitro* and two *in vivo* mouse model studies. Medical applications of NTP are being introduced, highlighting its great potential and hopefully helping to reverse worst-case scenarios.
